# Polycomb Repression without Bristles: Facultative Heterochromatin and Genome Stability in Fungi

**DOI:** 10.3390/genes11060638

**Published:** 2020-06-09

**Authors:** John B. Ridenour, Mareike Möller, Michael Freitag

**Affiliations:** Department of Biochemistry and Biophysics, Oregon State University, Corvallis OR 97331, USA; ridenojo@oregonstate.edu (J.B.R.); moellmar@oregonstate.edu (M.M.)

**Keywords:** *Cryptococcus*, fungi, *Fusarium*, histones, lysine methylation, *Neurospora*, polycomb repressive complex, PRC2, *Zymoseptoria*

## Abstract

Genome integrity is essential to maintain cellular function and viability. Consequently, genome instability is frequently associated with dysfunction in cells and associated with plant, animal, and human diseases. One consequence of relaxed genome maintenance that may be less appreciated is an increased potential for rapid adaptation to changing environments in all organisms. Here, we discuss evidence for the control and function of facultative heterochromatin, which is delineated by methylation of histone H3 lysine 27 (H3K27me) in many fungi. Aside from its relatively well understood role in transcriptional repression, accumulating evidence suggests that H3K27 methylation has an important role in controlling the balance between maintenance and generation of novelty in fungal genomes. We present a working model for a minimal repressive network mediated by H3K27 methylation in fungi and outline challenges for future research.

## 1. Introduction

Even though the first description of different chromatin types was cytological [1], transcriptional activity is frequently used to divide chromatin into three, sometimes four, categories that are correlated with post-translational modifications on histone tails [2]. Euchromatin is characterized by active transcription, and some examples of tell-tale histone marks are high levels of acetylation, e.g., on histone H3 lysine residues 9 and 27 (H3K9ac, H3K27ac), and di- or trimethylation of histone H3 lysine 4 or 36 (H3K4me2/3, H3K36me2/3). Constitutive heterochromatin lacks transcription of protein-coding genes, but non-coding RNA, either short siRNA or various types of long non-coding RNA (lncRNA) are produced from these regions, which are enriched in di- or trimethylation of histone H3 lysine 9 (H3K9me2/3) and methylated cytosines. A subtype of constitutive heterochromatin is “centrochromatin”, regions that contain the centromere and on which kinetochores form (reviewed in [3]). In many species, a histone H3 variant, CENPA (Cse4, Cid, CenH3), generates special nucleosomes that co-occur with canonical nucleosomes in centrochromatin [4]. The last type of chromatin is facultative heterochromatin, which typifies regions where transcription is reversibly turned on and off, either during responses to the environment, or during cell differentiation and development. The most intensely studied histone mark correlated with facultative heterochromatin is trimethylation of histone H3 lysine 27 (H3K27me3) [5,6]. This review is on facultative heterochromatin in fungi; we will largely focus on the past five years as several reviews have summarized earlier studies [7,8,9,10]. 

Compared to the breadth of information amassed in plants and animals (see recent reviews for summaries, e.g., [11,12,13,14,15,16,17,18,19]), the study of facultative heterochromatin in fungi can still be considered an immature field. Even though this form of heterochromatin had been recognized as important for differentiation and development for some time, it took until 2008 for the first demonstration of the presence of H3K27me3 in a fungus, the ascomycete *Neurospora crassa* [20]. Since then, our understanding has grown considerably, driven by comparative biology, and utilizing several key model organisms among the large and varied kingdom fungi, namely the saprobe *N. crassa*, the plant pathogens *Dothistroma septosporum*, several *Fusarium* species, *Magnaporthe oryzae*, *Verticillium dahliae, Zymoseptoria tritici*, the plant endosymbiont *Epichloë festucae*, and the facultative animal pathogen, *Cryptococcus neoformans*. 

Here, we will briefly summarize what has been found in studies on the single histone H3K27 methyltransferase complex, polycomb repressive complex 2 (PRC2), review effects on development and pathogenicity in the absence of fungal PRC2, and discuss how the interplay of various histone marks affects genome plasticity and stability in fungi. We propose a testable working model predicting the minimal components of a reversible H3K27me3-mediated gene silencing system in fungi, touch on ongoing forward genetic screens that promise to discover novel components of the system, and present an outlook describing several key challenges in the area of facultative heterochromatin in fungi for the immediate future.

## 2. Setting the Stage: Reverse Genetics to Define Facultative Heterochromatin in Fungi

Polycomb group (PcG) proteins were discovered by their impact on *Drosophila* development [21], and it took ~25 years before the molecular basis for their importance became known (reviewed in [5,17]). The realization that the PcG proteins, E(z), Su(z)12, and Esc, were required for gene silencing came early and resulted in the development of “chromatin immunoprecipitation” (ChIP) [22]. The dominant *Drosophila* Pc mutant, first isolated in 1947 by P. Lewis, received its name because it altered the number of sex combs, a row of thick bristles, on the legs of male flies (reviewed in [23]). Usually sex combs are found only on the first pair of legs, but Pc mutants have sex combs on all three pairs. E. Lewis realized that this body plan transformation resulted from a requirement of Pc to repress homeotic genes of the Bithorax complex [21]. Biochemical studies showed that PcG proteins fall into two main complexes, polycomb repressive complex 1 and 2 (PRC1 and PRC2), the latter of which included E(z), Su(z)12, and Esc ([12,23]. Subsequent studies identified human homologs (EZH2, SUZ12, and EED, respectively) that catalyzed H3K27 methylation [24,25] and additional subunits, including RbAp48/Nurf55 (called Msi1, Cac3, Msl1, or NPF in different fungi) that co-purified with the core PRC2 ([12,23]. Interest among researchers studying fungi increased when H3K27me3 was found in *Neurospora* [20]; notably, this important histone mark is absent in both budding and fission yeast [10,23]. 

Although not easily discerned by overall sequence comparisons, homologs of core PRC2 components found in fungi have well-conserved domain architectures when compared to their plant and animal counterparts [8,10,26] (Table 1). Crystal structures of a partial core PRC2 of the ascomycete, *Chaetomium thermophilum*, helped define functional regions of E(z)/EZH2 and Esc/EED homologs, and revealed structural similarity between fungal and animal PRC2s [26,27,28,29,30,31], while biochemical studies by a combination of western analyses, ChIP-seq, and mass spectrometry have demonstrated that PRC2 in fungi methylates H3K27 [32,33]. The most striking dissimilarities are the presence of a large “insertion domain” in fungal EED proteins that is missing from plant and animal proteins; the domain structure and boundary is conserved but the primary amino acid sequence is taxon-specific [10]. SUZ12 homologs are largely different, except for the VEFS motif, whose presence is difficult to detect in some fungi; SUZ12 may be supplanted by functional homologs in some fungi, e.g., in *C. neoformans* [34]. 

Reverse genetics studies delineated the PRC2 of fungi in the ascomycetes, *Neurospora* [35] and *Fusarium* [32], and the basidiomycete, *Cryptococcus* [34]. *Neurospora* PRC2 was characterized by deletion of homologous genes for all three core subunits (SET-7/EZH2, EED, SUZ12), which abrogated all H3K27me3, while deletion of the gene encoding an associated subunit (NPF/CAC-3/MSL1) resulted in lower H3K27me3 levels in many interstitial regions and complete absence of H3K27me3 from subtelomeric regions [35]. In *Fusarium graminearum,* deletion of *kmt6/EZH2* resulted in complete loss of H3K27me3 [32], and the same is true for deletion of *eed* or *suz12*, while deletion of *msl1/cac3* had no effect on H3K27me3 levels (Connolly and Freitag, unpublished results). Loss of the *C. neoformans* PRC2 core components (Ezh2 and Eed1) or the non-canonical subunit, Bnd1, removed all H3K27me3, while loss of Msl1 or the novel accessory component Ccc1 resulted in regional reduction and relocalization of H3K27me3 [34]. This was the first study that delineated fungal PRC2 components by biochemical means, and it identified two novel fungal PRC2 subunits that may be functional homologs for SUZ12 (e.g., Bnd1) or connect PRC2 to histone deacetylase (HDAC) complexes (e.g., Ccc1). In contrast to the findings with *Neurospora*, *Cryptococcus msl1* mutants showed only depletion of H3K27me3 in subtelomeric regions; *ccc1* mutants showed a more pronounced reduction of H3K27me3 in these regions.

**Figure 1:**
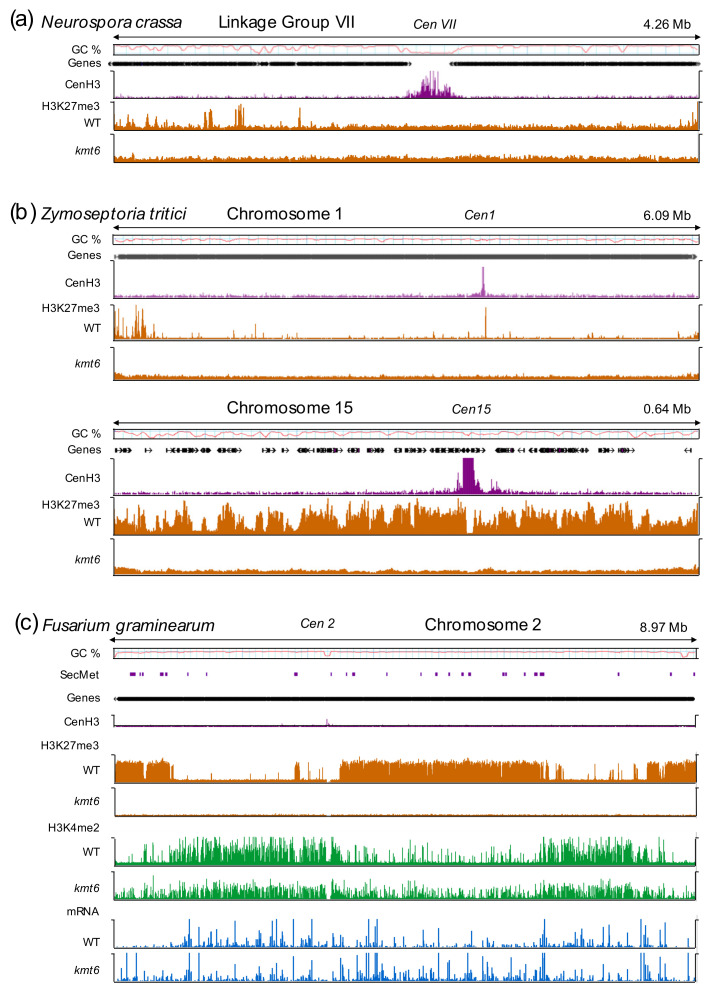
**Chromatin landscapes in selected filamentous fungi**. (**a**) In *N. crassa*, H3K27me3 covers subtelomeric regions and discrete interstitial sequences along the chromosome arms. Deletion of *kmt6* results in complete loss of H3K27me3. (**b**) H3K27me3 enrichment in *Zymoseptoria tritici* differs between core and accessory chromosomes. Core chromosomes (e.g., chromosome 1) show a similar distribution to *N. crassa* with enrichment in subtelomeric regions and local peaks. Conversely, accessory chromosomes (e.g., chromosome 15) are enriched with H3K27me3 over the entire length of the chromosome. Again, all H3K27me3 is lost upon deletion of *kmt6*. (**c**) *Fusarium graminearum* chromosomes are divided into large blocks of H3K27me3-enriched facultative heterochromatin and euchromatic regions, marked by H3K4me2. Loss of H3K27me3 increases both H3K4me2 enrichment and transcription (mRNA; blue) in many, if not all, former facultative heterochromatic blocks. H3K27me3 regions contain secondary metabolite (SecMet) clusters and pathogenicity genes that are activated in the absence of H3K27me3. ChIP-seq experiments with the centromere-specific histone H3, CenH3 (CENPA), indicate centromeric regions, which are largely free of H3K27me3, except small amounts in some *Z. tritici* centromeres [36]. Publicly available data were used to construct this figure [32,33,37,38].

Studies in these three models, and more recently the wheat pathogen, *Zymoseptoria tritici* [36,37], defined the extent of H3K27me3 in fungal genomes by ChIP-seq, and revealed upregulation of genes covered by H3K27me3 in PRC2 mutants (Figure 1). Together, these studies demonstrated that fungi have a canonical or largely conserved PRC2 that appears to act as a general repressor of gene transcription, but they also suggested taxon-specific roles played by H3K27 methylation.

## 3. Maintenance or De Novo H3K27 Methylation—or Both?

DNA replication requires disassembly of nucleosomes and eviction of DNA- and chromatin-interacting proteins. While strand-specific mechanisms of histone recycling have been uncovered (reviewed in [13]), it is not fully understood how H3K27me3 is reestablished following passage of the replication fork. PRC2 writes and reads H3K27me3, and colocalizes at replication forks, where it can aid DNA replication [39,40,41,42], suggesting a model of maintenance wherein PRC2 reestablishes H3K27me3 domains using recycled, H3K27me3-modified histones as a guide [43]. Recently, chromatin assembly factor 1 (CAF-1) was found to mediate the interaction between PRC2 and proliferating cell nuclear antigen (PCNA), a central component of DNA replication, in plants and animals [44,45]. In fungi, Msl1 is a component of CAF-1 in addition to its role as an accessory component of PRC2 [46]. PKL, a plant chromatin remodeling factor (CRF), promotes recycling of H3K27me3-modified histones following DNA replication [47]. Interactions among PRC2, CRFs, and DNA replication machinery have not been explored in fungi.

**Figure 2:**
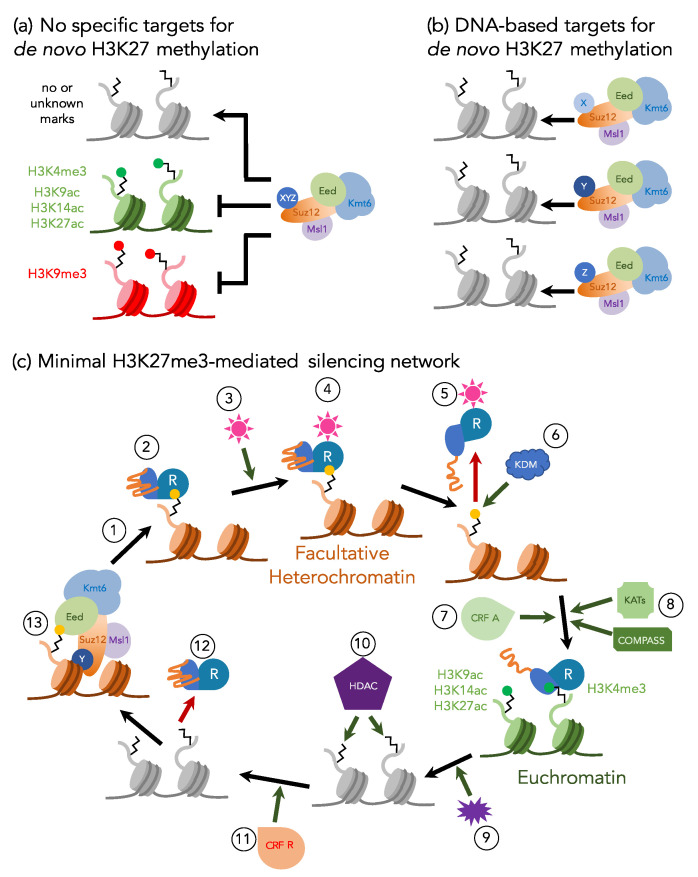
**Working models for *de novo* H3K27 methylation and a minimal H3K27me3-mediated silencing network**. (**a**) *De novo* H3K27 methylation in fungi may occur in all regions not blocked by proteins or specific histone modifications (“default H3K27me1/2 model”) as suggested by others [9,17,34,64]. (**b**) A non-exclusive model predicts recognition of specific nucleic acid-based signals by chromatin-binding proteins (X, Y, and Z) that recruit the core PRC2 to specific genes or blocks of genes, presumably by using SUZ12 as an adaptor, thus yielding a diverse population of PRC2-adaptor complexes. (**c**) Based on the combination of data from fungi and plants (e.g., western blots, ChIP, histone binding assays, phenotyping, and effects of mutants that have been isolated), we propose that an adaptor protein (e.g., *N. crassa* EPR-1 [58], *F. graminearum* DIS2; unpublished results from our lab), which is similar to *Arabidopsis* SHL [60] and EBS [61], acts as a “Reader” (R) for both silent and active chromatin regions. PRC2 catalyzes H3K27me3 either *de novo* or by close association with the replication fork (1), and the Reader binds H3K27me3 (yellow dot) in facultative heterochromatin via its bromo-adjacent homology (BAH) domain (teal; 2). Exogenous or endogenous cues (pink star; 3) may result in binding to (4) and conformational changes (5) of the Reader. One prediction is that the Reader is temporarily removed from the H3 tail, while a histone demethylase (KDM; 6) removes H3K27me3; an alternative model suggests that the Reader remains bound to H3K27me3 until H3K4me3 binding is possible after action of the H3K4 methyltransferase, COMPASS (8) [10]. Conformational changes within the Reader’s carboxy-terminal motif (orange tail) makes the plant homeo domain (PHD) (blue) accessible, which allows Reader binding to H3K4me3, likely in combination with the activity of activating chromatin remodeling factors (CRF A; 7) and lysine acetyltransferases (KATs; 8) that may acetylate H3K9, H3K14, or H3K27, or a combination of them (green dot). Gene expression is altered based on unknown exogenous or endogenous cues (purple; 9). The Reader may directly or indirectly recruit histone deacetylase (HDAC; 10) complexes, which—in combination with repressive chromatin remodeling factors (CRF R; 11)—aid in Reader release from H3K4me3 (12). Chromatin-binding adaptors bound to core PRC2 re-methylate regions destined to be facultative heterochromatin (13).

Contrasting evidence suggests that recycled H3K27me3 is not necessarily sufficient or required for long-term propagation of transcriptional repression. For example, in animals, lncRNAs have been suggested to target PRC2 to specific regions [17,48], something that has not been shown or even seriously explored in fungi. Specific DNA sequences or regions with certain physical characteristics, like polycomb response elements (PREs) in *Drosophila* [23,49,50] and hypomethylated CpG islands in mammals [51,52,53,54,55], recruit PRC2 and mediate transcriptional repression. Although similar mechanisms for H3K27me3 nucleation have not been identified in fungi, recent work in *N. crassa* showed that telomeric repeats can recruit H3K27me3 to naïve chromosome segments and spread H3K27me3 across dozens of kilobases [56]. Furthermore, depletion of H3K27me3 by mutation of PRC2 subunits can be faithfully reestablished following complementation of the causal mutation in fungi and animals [32,35,55]. From studies in mammals it is clear that H3K27me2 is wide-spread in chromatin (60–80% of all nucleosomes with H3), which suggested a “hit-and-run” activity (Figure 2) of PRC2 to generate H3K27me1/2; however, H3K27me3 activity may be more targeted. Together, this demonstrates that *de novo* PRC2 activity, likely conferred by sequence-specific binding, DNA structure, or an “epigenetic” assembly of RNA and protein factors, and maintenance activity work together to propagate H3K27me3-mediated repression in a manner dependent on the organism and the locus.

## 4. Defining the Minimal H3K27me3 Network: Fungi as Excellent Genetic Models

Fungi are excellently suited for genetic analyses, and studies in at least three laboratories are under way to determine what other components are part of the H3K27 methylation network. Two recent papers describe new components of the fungal H3K27 methylation network. A forward genetics selection with *N. crassa* identified a novel fungal-specific protein, PRC2 ACCESSORY SUBUNIT (PAS) [57]. PAS promotes H3K27me2/3 and transcriptional repression in subtelomeric regions, similar to what had previously been observed for NPF/MSL1 [35,57]. While additional studies will be required to determine the functional conservation of PAS or PAS-like proteins in fungi, preliminary results suggest that this protein is weakly conserved and may have taxon-specific functions.

Studies in our lab have identified several mutants, called *defective in silencing* (*dis*), which exhibit phenotypes that are near-identical to those observed with *kmt6* deletion strains. While our analyses are still incomplete and ongoing, one of these proteins, DIS2, resembles a protein also found recently in *N. crassa*, called EFFECTOR of POLYCOMB REPRESSION-1 (EPR-1) [58]. EPR-1 has a bromo-adjacent homology (BAH) domain followed by a plant homeo domain (PHD), both of which are able to bind modified or non-modified histone tails in other proteins [59]. In fact, the most similar two plant proteins, SHL [60] and EBS [61], have been proposed to bind H3K27me3 and H3K4me3 simultaneously (SHL) or sequentially (EBS), suggesting that this class of proteins may serve as a molecular switch from H3K4me3-enriched, active regions bound by the PHD, to H3K27me3-enriched, silent regions bound by the BAH domain [61]. The *N. crassa* EPR-1 protein may act in a similar fashion. All *epr-1* mutants display gene expression changes that are similar to strains lacking PRC2 components, but surprisingly H3K27 methylation appeared unaffected [58]. By cytology, EPR-1 clusters in subtelomeric foci, and ChIP-seq showed that its genome-wide distribution is dependent on H3K27me3-enriched chromatin [58].

Our working model predicts additional factors that are expected to be necessary to complete a feedback loop that establishes and maintains H3K27 methylation until environmental or internal conditions require transcriptional activation (Figure 2). Apart from PRC2 and various associated proteins, a PHD–BAH domain containing “Reader” can alternatively bind to H3K27me3 and H3K4me3, and serves as a switch from a silent to active state. Altering the Readers’ binding activity may require a histone demethylase (KDM) to remove H3K27me2/3, as well as histone lysine acetyltransferases (KATs) and the H3K4 methyltransferase COMPASS complex [62,63], both of which would be required to convert long-term silencing into long-term activation. Recruitment of KATs and COMPASS may also require the activity of chromatin remodeling factors (CRFs) to generate more open chromatin structures or even evict nucleosomes with silencing H3K27me3 marks. Once active gene expression is no longer required, external or internal stimuli recruit HDACs, perhaps again in combination with CRFs, which generate the proper conditions for PRC2 recruitment.

ATP-dependent CRFs catalyze nucleosome sliding, disassembly, and exchange, which ultimately influences transcription via nucleosome positioning or DNA accessibility [65,66]. Interplay among CRFs and PRC2 is emerging as an integral part of H3K27me3-mediated repression in plants. One such relationship is the genetic interaction between PKL, a CHD subfamily II-type CRF [67], and CLF, an H3K27 methyltransferase in a plant PRC2 [68]. PKL is involved in transcriptional regulation of developmental genes [69,70] and associates with H3K27me3-enriched loci [47,71]. In root tissue, the relationship between PKL and CLF appears to be antagonistic, as PKL promotes cell proliferation in the meristem in opposition to CLF, which restricts cell proliferation [72]. Loss of PKL repressed developmental genes and showed increased H3K27me3 levels [72]. In aerial tissue, evidence suggests that PKL cooperates with CLF and PIE1, an SWR1-type CRF that incorporates the histone variant H2A.Z, to promote H3K27me3 and transcriptional repression [73]. Moreover, H2A.Z appears to colocalize with H3K27 methylation in plants and animals [5,73]. It was recently demonstrated that in *N. crassa* H2A.Z and the CRF, SWR-1, similarly promote H3K27 methylation, although H2A.Z and H3K27 methylation were not found to colocalize [74].

Recent evidence from animal studies also suggests that CRFs have various roles in controlling PRC2 activity (reviewed in [75,76]). The nucleosome remodeling and deacetylase (NuRD) complex mediates transcriptional repression through nucleosome invasion and histone deacetylation and is proposed to promote PRC2 recruitment [77,78]. NuRD consists of a CHD subfamily II-type CRF similar to PKL (e.g., Mi-2 in *Drosophila*) and NURF55, a homolog of Msl1, among other components [79,80]. In contrast, the name-giving SWI/SNF CRF complex promotes open chromatin in opposition to PRC2 activity [81,82,83,84]. Mutations in SWI/SNF components are associated with regional increases in H3K27me3 levels and transcriptional repression [75,76]. Taken together, evidence suggests that NuRD and SWI/SNF act in opposition to modify the chromatin environment and subsequently promote or antagonize PRC2. All CRFs mentioned above have highly conserved homologs in fungi [85], and although little is known about these proteins in filamentous fungi, similar interplay among CRFs and PRC2 is expected to have an important role in H3K27me3-mediated repression.

## 5. Functions of H3K27 Methylation in Fungi

### 5.1. Regulation of Transcription

Generally, transcriptional activity is lower or absent in chromatin enriched with H3K27me3 [6]. Transcriptional repression has thus been long thought of as the most important, if not only, function of H3K27me3 and PRC2 in fungi. The most compelling evidence for transcriptional regulation and the important functions of PRC2 within the fungi come from studies with *Fusarium*, where development and differentiation are severely disturbed by the expression or overexpression of ~2,500 genes following loss of PRC2 [32]. In *N. crassa*, 700–900 genes are enriched with H3K27me3, and ~150 genes are derepressed in PRC2 mutants [33,35], but many of these transcripts emanate from regions that are not usually enriched with H3K27me3, suggesting an indirect effect on transcription. In *C. neoformans*, ~75 transcripts increase in expression by more than threefold, and 71% of these are within 40 kb of telomeric regions [34]. In *Z. tritici*, ~1,300 genes are enriched for H3K27me3, but only 98 genes in H3K27me3 regions and 124 genes in total were upregulated in absence of H3K27me3 [37]. While direct quantitative comparisons between these datasets are difficult, it is clear that H3K27me3 is involved in transcriptional regulation in fungi. For example, we fully expect that transcriptional activation in the absence of H3K27me3 requires additional chromatin reorganization or the activity of context-specific transcription factors, or both.

How is H3K27me3-dependent transcriptional repression mediated? After discovery of the molecular activity of PRC2, a hierarchical model of catalysis and spreading of H3K27me3 by PRC2 and subsequent binding of PRC1 by virtue of its Pc subunit, which reads H3K27me3, was proposed [24]. It is now clear that the hierarchical “PRC2 first, then PRC1” model cannot reconcile all available data from all organisms that have been studied, and it has thus been rejected. Even in animals not all PRC1 variants contain chromo domain or “chromobox” (Cbx) proteins. PRC1 subunits RING1A/B form distinct subcomplexes (PRC1.1 to PRC1.6); only PRC1.2 and PRC1.4 contain a Cbx [86,87,88]. Lack of PRC2 has only mild effects on global PRC1-mediated ubiquitination levels of H2A lysine 119 (H2AK119ub1) [89], and some PRC1s are recruited independently of PRC2 and H3K27me3 [90]. For example, the histone demethylase, KDM2B, can mediate PRC1.1 recruitment via its CXXC motif, which can bind unmethylated CG-rich DNA [91,92,93], which in turn may recruit PRC2. H2AK119ub1 then modulates binding and catalytic activity of PRC2 through interaction with an accessory subunit, JARID2 [88,94,95], which suggests a feedback loop involving both canonical PRCs. 

While many fungi have genes for the core PRC2 subunits, no fungal genome examined so far encodes known critical components of the canonical PRC1. There is no evidence for clear homologs of many PRC1 subunits, including KDM2B and JARID2, in fungal genomes. One catalytic activity of PRC1 is ubiquitination of H2A (H2AK118ub1 in flies, H2AK119ub1 in mammals, and H2AK121ub1 in plants), which is thought to result in transcriptional repression (reviewed in [13,17,48,96]). However, this model has not been investigated in any detail in fungi, simply because true Pc proteins are lacking from both fungi and plants [97,98]; thus, all facultative heterochromatin generated by “polycomb” PRC1 complexes must be generated by different mechanisms. In plants, the role of Pc may be at least partially fulfilled by LIKE-HETERCHROMATIN PROTEIN-1 (LHP1/TLF2), which functions in both PRC1 and PRC2 [98,99], but not in H3K9 methylation where canonical HP1 is required for normal function in both animals and fungi [100,101]. LHP1 promotes H3K27me3 spreading and maintenance via its function as H3K27me3 reader and its interaction with the plant Nurf55 homolog, MSI1 [98]. Another plant-specific protein, EMBRYONIC FLOWER-1 (EMF1), has been proposed to mediate H3K27me3-dependent silencing through chromatin compaction as a functional homolog for animal Psc proteins, and while EMF1 had been shown to interact with LHP1 *in vitro*, a recent study showed interactions with the BAH-domain containing H3K27me3 readers, SHL and EBS, in a BAH–EMF1 Complex to read H3K27me3 and induce transcriptional silencing [102]. In fungi, neither a chromo domain protein functionally similar to Pc or LHP1 nor a functional homolog of EMF1 have been found so far. Thus, it remains a complete mystery how exactly H3K27me3-mediated transcriptional repression is carried out in fungi; deciphering downstream effects of H3K27me3 on RNAP II activity will be an important goal for the next decade.

### 5.2. The Role of Facultative Heterochromatin During Development and Pathogenesis

Transcriptional regulation of important genes is considered the proximal cause for the requirement of PRC2-mediated H3K27 methylation for normal development in the plants and animals that have been studied. While the number of fungi, including plant pathogens, with experimental evidence for the presence of H3K27 methylation is rapidly increasing, the functional characterization of facultative heterochromatin is lagging behind. Loss of H3K27 methylation can slow growth and development in some fungi, most notably in the genus *Fusarium*. For example, deletion of genes encoding the core *F. graminearum* PRC2 subunits, *kmt6/EZH2* [32], *eed*, or *suz12* (Connolly and Freitag, unpublished results) resulted in severe defects in both linear growth and development. Colony morphology is severely disturbed, presumably because various pathways involved in signaling and asexual growth are expressed at improper times. Sexual development is completely abrogated in *kmt6* mutants under normal conditions in this homothallic species [32]; in outcrosses with engineered strains *kmt6* mutants are female sterile. Overexpression of numerous secondary metabolite clusters in *kmt6* mutants may contribute to defects in development and pathogenicity [38]. In the rice pathogen, *F. fujikuroi*, attempts to delete *kmt6* have been unsuccessful [103]. This suggests that PRC2-mediated gene silencing is essential for survival in this, and perhaps other, species. Quelling of *kmt6* by introduction of constructs to generate siRNA from double-stranded RNA revealed severe defects in growth and sporulation [103]. Further work will delineate why PRC2 components are essential in some *Fusarium* species but not others.

In plant pathogens other than *Fusarium*, deletion of *kmt6/EZH2* resulted in only minor defects in development. In the wheat pathogen *Z. tritici*, strains lacking H3K27me3 were indistinguishable from wild type in growth and stress responses (e.g., temperature, cell wall, osmotic, oxidative, genotoxic stress) except for a slight increase in melanization at elevated temperatures; pathogenicity on wheat was only mildly affected [37]. Deletion of *kmt6* in the blast pathogen, *Magnaporthe oryzae*, abolished all H3K27me3, reduced asexual sporulation, and resulted in severe defects in pathogenicity on wheat and barley [104]. *Epichloë festucae* is a beneficial endosymbiont of perennial ryegrass (*Lolium perenne*). Deletion of *ezhB*/*EZH2* abolished all H3K27me3 [105]. This fungus is well known for production of ergot alkaloids and indole diterpenes, which afford the host plant protection from insect and mammalian herbivory [106]. Genes underlying the production of ergot alkaloids and indole diterpenes are organized in the subtelomeric gene clusters, *eas* and *ltm*, respectively, and only expressed *in planta* [106,107]. *In vitro* suppression of *eas* and *ltm* genes was influenced by presence of heterochromatic marks, i.e., H3K9me3 or H3K27me3 [105]. H3K9me3 and H3K27me3 levels at *eas* and *ltm* were reduced *in planta* when compared to *in vitro* growth. Deletion of the H3K9-methyltransferase-encoding gene, *clrD*, or *ezhB* increased expression from both clusters *in vitro*, and double deletion resulted in additive effects [105]. However, *in vitro* metabolite production was not observed in any of the mutants [105]. Loss of EzhB on ryegrass resulted in increased expression of all tested genes in the *eas* cluster, including genes that were not derepressed *in vitro*, and increased production of the ergot alkaloid, ergovaline [105], suggesting a host-derived signal is required even in the absence of H3K27me3 repression. Strains lacking EzhB effectively colonized ryegrass yet negatively impacted plant development, while deletion of *clrD* abolished colonization of ryegrass [105], demonstrating that H3K9 and H3K27 methylation are somehow required for establishing and maintaining successful symbiosis. Dothistromin, a secondary metabolite produced from a developmentally-regulated pathway in the pine needle blight pathogen, *Dothistroma septosporum*, is regulated by H3K27me3 [108]. During development, dothistromin cluster genes were transcribed early and later repressed by H3K27me3. Although dothistromin is a key virulence factor in *D. septosporum*, the impact of *kmt6* deletion on pathogenesis has not been determined [108]. 

Overt phenotypes are not observed in *N. crassa* or *C. neoformans* in the absence of H3K27me3 under normal laboratory growth conditions, but loss of accessory components, e.g., NPF/CAC-3/MSL1 in *Neurospora* and Msl1 or Ccc1 in *Cryptococcus*, resulted in minor growth reduction, presumably because these subunits are also found in several other complexes involved in chromatin regulation [34,35]. The important animal pathogens, *Candida albicans* and *Aspergillus fumigatus*, lack core PRC2 components and no H3K27 methylation has been detected [10]. So far, *C. neoformans* and *Fusarium oxysporum* are the only animal pathogens among the fungi in which H3K27me3 has been demonstrated [34,109], even though the role of facultative heterochromatin in pathogenesis has not been directly studied. In *Cryptococcus*, Msl1 is required for multiple virulence-associated phenotypes, including thermotolerance, cell wall stability, and melanin production, as well as full virulence in a murine infection model [46]. Loss of Hda1 causes defects in multiple virulence-associated phenotypes similar to *MSL1* deletion, reduced survival in murine macrophages, and decreased virulence in a murine infection model [110]. These results emphasize the importance of determining the extent to which PRC2 influences pathogenesis. Taken together, all available evidence suggests that H3K27me3 has roles in growth and development under certain conditions in all fungi that have been studied, though the effects have been most obvious in the genus *Fusarium*. Furthermore, functional studies in pathogenic or symbiotic fungi have generally focused on specific and regional effects, so direct evidence for genome-wide chromatin changes during host infection remains scant.

### 5.3. H3K27me3 as a Driver for Genome Plasticity

Genome integrity is essential for the maintenance of cellular function and viability. Genome instability is a phenomenon that is frequently observed in malfunctioning cells associated with disease in many eukaryotes but has also been reported as advantageous for rapidly adapting organisms [111,112]. This suggests that genetic variability offers fitness advantages in certain environments that are selected for under specific conditions.

Pathogenic fungi are prominent examples of organisms with a high degree of genetic diversity [113]. Studies with human and plant pathogens showed extraordinary genome dynamics upon exposure to environmental stressors (e.g., fungicides) resulting in adaptation and increased fitness [114,115,116,117]. An important challenge in understanding genome dynamics is to elucidate mechanisms that confer or promote genetic variability. 

**Figure 3:**
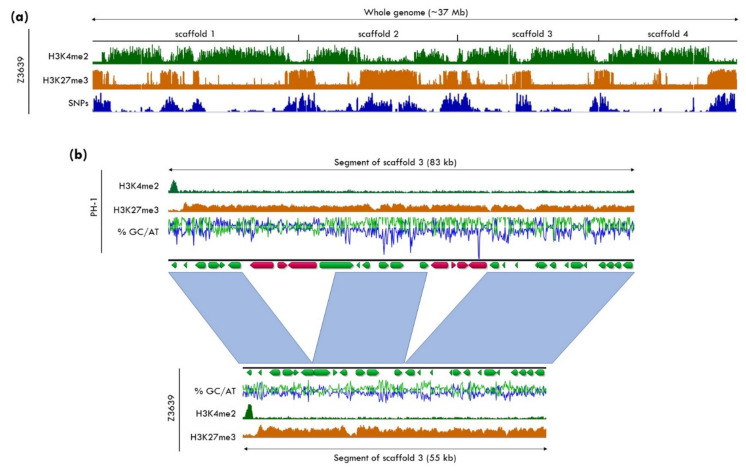
**Variability of H3K27me3 regions in different *Fusarium graminearum* isolates**. (**a**) The genome of *F. graminearum* isolate Z3639 consists of four chromosomes (scaffolds 1–4) that are largely divided into blocks of euchromatin, marked by H3K4me2, and facultative heterochromatin, marked by H3K27me3. Genome comparison between the two newly completed *F. graminearum* genomes from two closely related isolates, Z3639, and the reference strain, PH-1 [118,119], revealed increased SNP density in H3K27me3-marked regions compared to euchromatic regions of the genome, as previously suggested [32,118]. (**b**) Sequence comparison within a large H3K27me3-enriched region between PH-1 and Z3639 shows that overall synteny between H3K27me3 regions is largely conserved between isolates but insertions and deletions of genes are frequently found in these regions. Note that scaffolds are arranged by size, so that “scaffold 3” refers to chromosome 4 and “scaffold 4” refers to chromosome 3 of previous genome assemblies. Complete genomes of Z3639 and PH-1 were sequenced and assembled in collaboration with the JGI, and complete ChIP-seq data sets from Z3639 will be part of a separate publication (Ridenour, Möller, and Freitag, in preparation).

Population genetic studies demonstrated that DNA sequences subject to rearrangements and instability are not randomly distributed within the genome, but rather specific regions show higher variability than others. Sequences located close to chromosomal ends, the subtelomeric regions, and sequences harboring active or mutated transposable elements (TEs) show a high degree of diversity, even between isolates of the same species [120,121,122]. These differences include a higher density of SNPs, presence/absence polymorphisms of genes and TEs, or structural rearrangements (Figure 3). Furthermore, various fungal plant pathogens contain conditionally dispensable, “accessory” chromosomes or sections of chromosomes, that, in contrast to the “core” genome, are present in only some isolates and sometimes of benefit to the species under certain environmental conditions [123,124,125]. Often, variation in these regions or chromosomes affects genes that are involved in rapid adaptation, e.g., pathogenicity factors or biosynthetic gene clusters that are required for the production of secondary metabolites [126,127,128,129].

Chromatin organization differs between highly variable regions and the more conserved core regions of genomes, and all evidence shows that H3K27 methylation is enriched in subtelomeric regions and on accessory chromosomes (Figure 1). There is mounting evidence linking both constitutive and facultative heterochromatin and genome organization within nuclei of filamentous fungi. Hi-C, a method to investigate three-dimensional genome organization, showed that in *N. crassa*, a mutant karyopherin (DIM-3) involved in the assembly of constitutive heterochromatin changed average chromosome structure [130], while a *set-7/kmt6* deletion mutant resulted in the movement of subtelomeric regions to the interior of the nucleus and occasional disruption of centrochromatin, measured by the appearance of several CenH3 foci, rather than the usual single focus [131], in each nucleus [130,132]. Hi-C and RNA-seq were also used to study how the genome structure of *E. festucae* contributes to symbiosis with ryegrass [133]. Repetitive, AT-rich regions were considered to be in a more condensed chromatin state, and genes differentially expressed *in planta* were overrepresented near these regions. For example, the *eas* gene cluster (see Section 5.2) was found interspersed between AT-rich regions in a single topologically associated domain (TAD) *in vitro* [133], supporting the idea that facultative heterochromatin and genome organization have a role in regulating gene expression in a context-specific manner.

A series of studies documented that H3K27me3 is critical for conferring genome instability, which contributes to high genetic variability. In *Z. tritici*, H3K27me3-marked accessory chromosomes are highly unstable during both meiosis and mitosis; they are frequently lost from individuals but maintained in the population as a whole [36,117,134,135]. In the absence of H3K27me3 in *kmt6* deletion mutants, the mitotic loss rate of accessory chromosomes decreased ~4-fold, suggesting a destabilizing effect caused by the presence of H3K27me3 [37]. Reintroduction of H3K27me3 in complementation strains restored loss rates back to the relatively high levels observed in the wild type (~7% of tested cells). Increased loss rates of an accessory chromosome, likely caused by increased H3K27me3 enrichment, were also reported in *F. fujikuroi* mutants that lacked the H3K36 methyltransferase, Ash1, supporting the hypothesis that the presence or imbalance of H3K27me3 decreases stability (the connection between methylation of H3K36 and H3K27 is discussed in Section 5.4) [136]. 

An imbalance of H3K27me3 as a consequence of loss of other histone marks that exclude H3K27me3 from specific chromosomal regions under standard lab conditions has been previously shown in *N. crassa* and *C. neoformans*. Genetic analyses revealed a genetic connection between constitutive and facultative heterochromatin in *N. crassa* [33]. Mutants defective in the H3K9 methylation system experience genotoxic stress, for example caused by methyl methanesulfonate (MMS) [137,138]. A selection scheme to isolate suppressors of this phenotype resulted in the identification of a deletion mutant of the PRC2 subunit, EED [33]; similar results were obtained by crossing existing single deletion strains of DIM-5/Kmt1 and SET-7/Kmt6 [139]. ChIP-seq analyses showed that H3K27me2/3 moved into regions previously occupied by H3K9me3 [33,139]. If only HP1 was lacking, constitutive heterochromatin was enriched with both H3K9me3 and H3K27me3 [139]. Mutants deficient in both H3K9 and H3K27 methylation rescued overt phenotypes, but accumulated γH2A and became more sensitive to camptothecin, a topoisomerase I inhibitor [33,139]. Combined, these results suggested that gaining H3K27me2/3 in regions that are usually enriched with H3K9me3 did not result in compensation for silencing of transposons or transcriptional dysfunction in regions that are usually occupied by H3K27me3, but rather that PRC2 modulates genotoxic stress genome-wide; almost all known transposons in *N. crassa* are inactivated by repeat-induced point mutation (RIP), which attracts H3K9me3, and, in turn, allows cytosine DNA methylation [140,141]. Similar results were obtained in *C. neoformans*. Mutants of *ezh2/kmt6* and the novel *ccc1* subunit were tested for conditional relocalization of H3K27me3 to constitutive heterochromatin; H3K27me3 moved into regions of constitutive heterochromatin in *ccc1* mutants [34]. The authors proposed that binding of Ccc1 to H3K27me3 restricts relocalization, while in absence of Ccc1, EED binds to H3K9me3 resulting in H3K27me3 enrichment in H3K9me3 regions [34]. However, in *N. crassa*, the presence of H3K9me2/3 is not necessary for H3K27me3 relocalization, suggesting additional factors involved in this process. Recent reviews proposed models (see Figure 2) to explain these data [8,9].

Recent work in *Z. tritici* came to similar conclusions [37]. Loss of H3K9me3 in *kmt1* deletion strains resulted in redistribution of H3K27me3 to regions that are normally covered by H3K9me3 [37]. Sequences that acquired H3K27me3 were shown to be involved in genome rearrangements in an evolution experiment that monitored genome stability in H3K27me3- and H3K9me3-deficient strains. However, in contrast to the situation in *N. crassa*, there was no increase in sensitivity to MMS in *kmt1* deletion mutants, suggesting that even wild-type *Z. tritici* strains are exquisitely sensitive to MMS, at much lower concentrations than *N. crassa*, and that genotoxic effects are therefore difficult to compare. The chromosome loss in *kmt1* deletion strains was twice as high as in the wild type, suggesting that relocalization of H3K27me3 resulted in genome destabilization, while removal of the redistributed H3K27me3 has a genome stabilizing effect because chromosome loss in the *kmt1/kmt6* double deletion mutant was decreased to wild-type levels [37].

### 5.4. Interaction of H3K27me3 with Other Chromatin Marks

So far, relatively little is known about interactions of H3K27me3 with other chromatin marks and modifiers in fungi, but studies in flies [142] and mammals [143] demonstrated that H3K36 methylation can antagonize PRC2-mediated H3K27 methylation. In budding yeast, all H3K36 methylation is catalyzed by a single KMT, Set2, and while H3K36me3 is generally correlated with regions that are actively transcribed, the molecular effect of H3K36me3 is transcriptional repression [144]. Set2 associates with RNAP II and is involved in transcript quality control during both initiation and elongation [145]. In fission yeast, H3K36me3 reduces overall chromatin accessibility and promotes non-homologous end joining (NHEJ) in a cell cycle-specific manner [146]. A *Neurospora set-2* mutant appeared to lack all H3K36 methylation by western blots, showed poor growth, poor conidiation, and was female sterile; an H3K36L substitution recapitulated these defects [147]. In *F. graminearum*, ChIP-seq showed that H3K36me3 was enriched at every single gene; euchromatic genes had H3K36me3 in 3’ regions of transcripts, while genes with H3K27me3 enrichment had H3K36me3 across the entire gene and the promoter [32].

Two recent studies in *F. fujikuroi* [136] and *N. crassa* [148] addressed the role of H3K36 methylation in transcriptional regulation, genome stability, and the relationship between H3K36me2/3 and facultative heterochromatin marked by H3K27me2/3. Many plants, fungi, and animals have additional methyltransferases involved in RNAP II-independent H3K36me2/3 [149]. In combination, *N. crassa* and *F. fujikuroi* Set2 and Ash1 are responsible for all H3K36me2/3. While the RNAP II-dependent Set2 marks euchromatic regions, Ash1-catalyzed H3K36me2/3 overlaps with poorly transcribed regions, specifically subtelomeric regions [148]. In the case of *F. fujikuroi*, Ash1-catalyzed H3K36me3 overlaps accessory chromosomes that are also characterized by the presence of H3K27me2/3 [136]. Loss of Ash1-, but not Set2-mediated H3K36me3, affected H3K27me2/3. In *F. fujikuroi*, deletion of Ash1 and subsequent regional loss of H3K36me2/3 increased H3K27me3 levels in several subtelomeric locations. Similarly, in *N. crassa*, loss of Ash1-mediated H3K36me2/3 enabled H3K27me2/3 to spread to neighboring sequences. Notably, if loss of H3K36me2/3 was accompanied by gene activation, H3K27me2/3 was also lost in some regions.

In *F. fujikuroi*, subtelomeric regions and an accessory chromosome, normally covered by H3K27me3 and Ash1-mediated H3K36me3, became unstable in *ash1* deletion mutants as observed by chromosome breakage or even complete loss of the chromosome [136]. These findings indicate that RNAP II-independent H3K36 methylation is important to balance H3K27me2/3 levels, and that an increase and spread of H3K27me2/3 can have detrimental consequences for the cell.

Numerous studies in animals have addressed the interplay between 5-methylcytosine (5mC) and H3K27me2/3 distribution. H3K27me3 enrichment is reduced at normal target genes upon loss of 5mC, and relocalizes to loci with normally high 5mC levels [150], including imprinted genes [151], demethylated CpG islands (CGIs) [52], some TEs [152], and pericentric or centromeric regions [153], which can also involve PRC1 activity [95]. The major consequence that has been studied in animals is transcriptional reprogramming, which results in altered development or disease [154]. Similarly, H3K27me3 undergoes redistribution to demethylated transposons in *Arabidopsis*, mutants deficient for the CG-specific maintenance methyltransferase, MET1 [155]. One of the few accepted rules about recruitment of the two main PRC2 variants in animals is that they effectively bind unmethylated CGIs [17]. In *N. crassa,* the only fungus in which the relationship between 5mC and H3K27me3 has been investigated, loss of 5mC does not affect distribution of H3K27me2/3 [139].

So far, little is known about interactions of histone lysine acetylation (KAT) or deacetylation complexes, either HDACs or sirtuins, with PRC2. One of the two novel subunits of *C. neoformans* PRC2, Ccc1, is similar to proteins found in HDAC complexes and associates with the class II HDAC, Hda1 (Clr3); deletion of Hda1 results in reduced growth as observed for Ccc1 and de-repression of PRC2-silenced genes in subtelomeric regions [34,110]. While further work is clearly necessary, it is possible that PRC2/Ccc1 and HDACs co-operate in transcriptional regulation of developmental genes.

From this glimpse into connections between different chromatin marks, it is clear that our understanding of interactions between chromatin modifications and the function of H3K27 methylation is still rudimentary, and that it will take comparative studies in several organisms to uncover all existing connections.

### 5.5. Evolution of H3K27me3-Dependent Transcriptional Silencing from an Ancestral Immune System Function

Population genetic analyses as well as recent experimental work indicated that H3K27me3 is connected to sequence diversity and instability. An intriguing puzzle to solve in the near future is how H3K27me3 can confer instability on a molecular level, and why this mechanism evolved over evolutionary timescales. While functional studies on the molecular mechanism are still lacking, a recent study on early land plants suggested that H3K27me3 together with H3K9me3 might represent an ancient form of genome defense mechanism against transposons [156]. Regions enriched with H3K9me2/3 and DNA cytosine methylation are usually AT- and repeat-rich, and movement of H3K27me3 to these regions in absence of H3K9me3 hints to a compensatory genome defense mechanism that was previously proposed to act in animals [152]. Redundancy between PRC2 and the cytosine DNA methylation pathways ensures that transposons remain silent, yet the gain of H3K27 methylation in constitutive heterochromatin in *N. crassa* appeared deleterious rather than compensatory [8].

**Figure 4:**
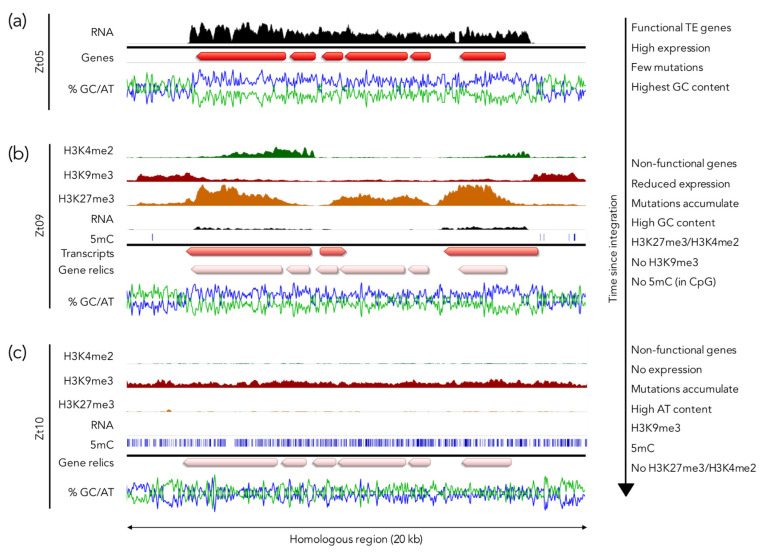
**H3K27me3 as part of an ancestral genome defense.** Homologous regions on different chromosomes in three *Z. tritici* isolates illustrate a model for H3K27me3-mediated silencing and step-wise inactivation of GC-rich regions on a short evolutionary timescale. (**a**) A recent transposon insertion in a subtelomeric region in the isolate Zt05. The GC-rich gene cluster containing transposon-related genes is flanked by AT-rich repetitive sequences. The genes inside the cluster are highly expressed. ChIP-seq and 5mC data are currently not available for this isolate. (**b**) A homologous region in the isolate Zt09 is marked by H3K27me3 and H3K4me2 enrichment in the GC-rich region and H3K9me3 in the flanking AT-rich regions. Compared to Zt05, there are ~600 mutations, mainly transitions, in the GC-rich region. Some sequences are expressed but the expression level is relatively low, and the genes are likely non-functional due to the numerous mutations. Very few 5mC sites are present in H3K9me3 regions as this isolate is lacking the DNA methyltransferase Dim2 [162]. (**c**) The isolate Zt10 accumulated > 1000 mutations in a homologous region, decreasing the GC content to ~49% (~60% in Zt05). The entire region is covered by H3K9me3 and 5mC and there is no detectable transcriptional activity or H3K4me2. The isolate Zt10 contains a functional DNA methyltransferase Dim2 [162]. Data used to generate this figure are from [37,162,163], and Möller and Freitag, unpublished results.

While H3K9me3 regions in fungi are AT-rich, this is not necessarily the case for H3K27me3 regions (Figure 4). Furthermore, the presence of H3K9me3 universally indicates the presence of an already silenced and inactivated transposon, whereas integration of an active transposon (and sometimes associated “hitch-hiking” genes) is predicted to recruit H3K9me3 only to AT-rich, repetitive regions [157,158,159] but not potentially harmful GC-rich genes. By comparing genomes and chromatin marks in different *Z. tritici* isolates, several H3K27me3-marked, isolate-specific regions were identified (Möller and Freitag, unpublished results). These were often flanked by H3K9me3-enriched regions and contained genes that are most similar to transposons or their “cargo” (Figure 4a). In contrast to the AT-rich H3K9me3 regions, the internal H3K27me3-covered regions harbored sequences that were slightly more GC-rich than the genome average. These sequences were transcriptionally repressed yet not completely silenced. Comparison of homologous loci in different *Z. tritici* isolates led to the hypothesis that H3K27me3 may function as an initial mechanism to mark “foreign” GC-rich DNA sequences to initiate transcriptional repression (Figure 4b). Over time, these H3K27me3 regions accumulate additional, mostly transition mutations that completely inactivate the now silenced genes, and eventually the GC content becomes so low that an H3K9me3 deposition mechanism similar to that envisioned in *N. crassa* [140] takes over, which also allows for wide-spread 5mC deposition [160,161] (Figure 4c). Isolate-specific, H3K27me3 regions should therefore be considered as meta-stable regions that, likely based on selection on the underlying genes, become permanently inactivated, neo-functionalized due to increased mutation rates, or conditionally repressed due to so far unknown dynamics of H3K27me3 silencing. This, or a similar system, may operate in other species as well, but detailed comparative studies in multiple strains of known lineages are lacking for other species. Detailed mechanistic studies on *Z. tritici*, *F. graminearum*, and *N. crassa* with several completely sequenced isolates is finally possible, and all three species are amenable to genetic, biochemical, and cytological studies.

## 6. Open Questions to be Resolved in the Near Future

***How many different PRC2s exist in fungi?*** In animal cells there seem to be two core PRC2s (EZH1 or EZH2, plus EED and SUZ12) that result in the formation of a variety of subcomplexes [17]. In plants there are three EZH2 and three SUZ12 core subunits each that use a single EED homolog, yielding a minimum of three PRC2s [18]. These various complexes are expressed at different times and under varying conditions, having separate but partially overlapping functions. Biochemical studies have been carried out in *N. crassa* and *C. neoformans* but again only under standard laboratory growth conditions. Based on the number of putative DNA-binding “transcription factors” (from ~200 to ~700 in *N. crassa* and *F. graminearum*, respectively) and the increase in percentage of the genome enriched with H3K27me3 (from ~7 to ~32% in *N. crassa* and *F. graminearum*, respectively), we propose that there is an increased number of PRC2 adapter proteins that interact with SUZ12 to recruit PRC2 activity to its target regions in some fungi, e.g., *F. graminearum*. This large number of repressor complexes would be balanced by activator complexes that are coupled to HAT co-activator complexes.

***How does H3K27me3 and facultative heterochromatin control fungal development?*** In plants and animals, H3K27me3 is involved in differentiation and development but no comprehensive time course studies across a complete life cycle have been carried out in fungi. This is an important knowledge gap that must be filled in the near future.

***What happens to H3K27me3-enriched regions in pathogenic fungi in response to the host environment?*** We are largely naïve on this issue, as successful and routine *in planta* ChIP-seq is still extremely difficult. H3K27me3 covers more than 30% of the *F. graminearum* genome during growth *in vitro*, including regions enriched with pathogenesis-associated genes [32]. Many of the genes covered by H3K27me3 *in vitro* are derepressed *in planta* [32,164]; a similar situation has been observed in other pathogenic fungi (e.g., *F. oxysporum* and *Verticillium dahliae* [109,165]). This strongly suggests that fungal pathogens must redistribute H3K27me3 or relax transcriptional repression associated with H3K27me3 in response to the host environment to facilitate pathogenic development within the host. To enable future studies, we are designing a system that allows enrichment and purification of fungal nuclei from infected host plants, which will augment current efforts to determine how chromatin structure of plant pathogenic fungi changes in response to the host environment and how the host responds to this challenge.

***How are chromatin domains organized in three-dimensional space within the nucleus?*** Existing Hi-C studies revealed that facultative heterochromatin has major impacts on nuclear organization, and we are currently only at the beginning of these studies. Hi-C investigations in double and triple mutants that are defective for several chromatin marks at the same time are underway.

***How do we reconcile genome metastability, increase of point mutations, chromosome loss, and transcriptional effects at the evolutionary scale?*** Comparing existing evidence from all fungi demonstrates that transcriptional regulation is not the only role of PRC2. Using several strains of the same species and several species of the same genus is now possible in all four genera discussed here. Genome sequencing, resulting in complete genome assemblies, ChIP-seq, Hi-C, and RNA-seq, are now routine and no longer prohibitively expensive. We predict that numerous “lab evolution” studies will help to resolve the current uncertainties. To this end, we also have begun to investigate the mechanistic connections between spontaneous and induced DNA lesions, fixed mutations, replication, and H3K27me3 targeting. The surprising observation in *Z. tritici* suggesting that H3K27me3 generates a “metastable” genome makes this species an ideal model to uncouple transcriptional and genome stability effects in future studies.

## Figures and Tables

**Table 1:** **Components of polycomb repressive complexes (PRC) in fungi**. Known components from *Drosophila*, human, *Fusarium graminearum*, *Neurospora* crassa, *Cryptococcus neoformans*, and *Arabidopsis thaliana*. All fungi examined to date have single homologues of at least two core PRC2 subunits (KMT6 and EED). SUZ12 and PRC2-associated subunits, such as MSL1/NPF or PAS appear more specialized and may have impacts only on certain chromatin regions. While KDMs with similarity to JARID2 homologs, and RING proteins are present in fungi, PRC1 subunits or other PRC2-targeting complexes found in animals are not conserved in fungi.

*Drosophila*	*Human*	*Fusarium*	*Neurospora*	*Cryptococcus*	*Arabidopsis*
**Core PRC2**					
enhancer of zeste, E(z)	EZH2/EZH1	KMT6	SET-7	Ezh2	MEA/SWN/CLF
extra sex combs, Esc/Escl	EED3/1/2/4	EED	EED	Eed1	EED
suppressor of zeste 12, Su(z)12	SUZ12	SUZ12	SUZ12	none	EMF2/VRN2/FIS2
none	none	none	none	Bnd1	none
**PRC2-associated proteins**					
Nurf55	RBAP48/46	MSL1	NPF	Msl1	MSI1
polycomb-like (Pcl)	PCL1/2/3	None ^1^	none	none	none
Jarid2	JARID2	None ^2^	none	none	none
JING	AEBP2	none	none	none	none
none	none	none	none	Ccc1	none
none	none	PAS ^3^	PAS	none	none
**PRC1 and other PRC-targeting complexes (PhoRC)**					
polycomb (Pc)	CBX2/4/6/7/8	none	none	none	none
none	none	none	none	none	LHP1 (TFL2)
none	None ^4^	DIS2	EPR-1	Epr1	EBS/SHL
polyhomeotic (Ph)	PHC1/2/3	none	none	none	none
posterior sex combs (Psc)	BMI1/PCGF2	none	none	Bmi1	BMI1
none	none	none	none	none	EMF1
Sex combs extra (RING)	RING1B/A	none	none	none	RING1B/A
Sex combs on midleg (Scm)	SFMBT1	none	none	none	none
Pleiohomeotic (Pho), Zeste	YY1 ^5^	none	none	none	AG, AS1/2, ALs, VAL1/2, COLDAIR

^1^ Poor homologs to the PHD of Pcl are found in early diverging fungi (“zygomycetes”). ^2^ The only known JARID homologs are functional histone demethylases of the KDM5 type. ^3^ PAS proteins lack conserved motifs and are not widely conserved even in fungi. ^4^ Human BAHD1 has a BAH domain that is similar to that of DIS2 and EPR-1. ^5^ Numerous transcription factors and lncRNAs have been correlated with PRC2 action.
